# Mapping the *Anthocyaninless *(*anl*) Locus in Rapid-Cycling *Brassica rapa *(RBr) to Linkage Group R9

**DOI:** 10.1186/1471-2156-8-64

**Published:** 2007-09-25

**Authors:** Carrie Burdzinski, Douglas L Wendell

**Affiliations:** 1 Department of Biological Sciences, Oakland University, 2200 N. Squirrel Rd, Rochester, MI 48309-4401, USA

## Abstract

**Background:**

Anthocyanins are flavonoid pigments that are responsible for purple coloration in the stems and leaves of a variety of plant species. *Anthocyaninless *(*anl*) mutants of *Brassica rapa *fail to produce anthocyanin pigments. In rapid-cycling *Brassica rapa*, also known as Wisconsin Fast Plants, the anthocyaninless trait, also called non-purple stem, is widely used as a model recessive trait for teaching genetics. Although anthocyanin genes have been mapped in other plants such as *Arabidopsis thaliana*, the *anl *locus has not been mapped in any *Brassica *species.

**Results:**

We tested primer pairs known to amplify microsatellites in *Brassicas *and identified 37 that amplified a product in rapid-cycling *Brassica rapa*. We then developed three-generation pedigrees to assess linkage between the microsatellite markers and *anl*. 22 of the markers that we tested were polymorphic in our crosses. Based on 177 F_2 _offspring, we identified three markers linked to *anl *with LOD scores ≥ 5.0, forming a linkage group spanning 46.9 cM. Because one of these markers has been assigned to a known *B. rapa *linkage group, we can now assign the *anl *locus to *B. rapa *linkage group R9.

**Conclusion:**

This study is the first to identify the chromosomal location of an anthocyanin pigment gene among the *Brassicas*. It also connects a classical mutant frequently used in genetics education with molecular markers and a known chromosomal location.

## Background

Anthocyanins are flavonoid pigments that are responsible for purple coloration in the stems and leaves of a variety of plant species. They have been cited as contributing to protection from photoinhibition [1], protection from UVB light [2] and modification of captured light quality and quantity [3]. Additionally, anthocyanins may be involved in metal accumulation. For example, *Brassica *anthocyaninless mutants show decreased tungsten accumulation [4] and fail to produce a water-soluble blue compound, likely a molybdenum-anthocyanin complex, in peripheral cell layers upon addition of molybdenum [5].

Anthocyanin-related genes have been isolated in diverse species. *AN1 *and *AN2 *encode transcription factors that regulate pigment production in petunia [6]. *Pp1, Pp2 *and *Pp3 *contribute to purple pigment in bread wheat [7]. Anthocyanin accumulation in pepper flowers is attributed to gene *A*, orthologs of which have been mapped in the related *Solanaceae *species tomato and potato [8]. In the model organism *Arabidopsis thaliana*, study of anthocyaninless mutants led to the discovery of *ANTHOCYANINLESS1 *(TAIR locus ANL1), responsible for anthocyanin production; *ANTHOCYANINLESS2 *(*ANL2*), a homeobox gene that affects anthocyanin distribution [9]; *ANTHOCYANIN11 *(TAIR locus AT1G12910), which contributes to anthocyanin production; *TRANSPARENT TESTA 9 *(TAIR locus TT9), which is involved in flavonoid biosynthesis, and three other pigmentation genes (TAIR loci AT1G56650, AT5G13930, and LAB).

Anthocyaninless mutants of rapid-cycling *Brassica rapa *(RBr), also known as Wisconsin Fast Plants, completely lack the purple coloration. (Wisconsin Fast Plants is a trademarked name, so we shall refer to them as RBr.) RBr are strains of *Brassica *bred for short life cycle, early flowering, and ease of cultivation, and are used in science education and research [10]. Absence of anthocyanin pigment in RBr is a recessive trait controlled by the *anthocyaninless *(*anl*) locus. The anthocyaninless trait, also called non-purple stem, makes an excellent model trait in monohybrid crosses in genetics education because the trait is easily scored and expressed at all stages of the life cycle [11]. In addition to its use in education, the *B. rapa *anthocyaninless phenotype has also been used as a marker to assess honey bee pollen deposition patterns [12] and to evaluate gene flow from transgenic plants into wild relatives [13].

RBr are a valuable tool for "hands on" genetics teaching. Cultivation is simple and inexpensive, and genetic crosses are easy to perform because they are self-incompatible for pollination. Many easily scored Mendelian traits have been identified including anthocyaninless, yellow-green (yellow-green coloration of all leaves), and hairless (lack of trichome on stems and leaves), to name a few. In addition to Mendelian traits, quantitative and polygenic traits are also available. For example, in those plants that possesses a wild type *ANL *allele, the intensity of anthocyanin coloration is a quantitative trait described as *purple anthocyanin (0–9) *(*Pan(0–9)*), which is controlled by multiple modifying alleles [14,15]. Likewise, the trichome density, or "hairiness," is a quantitative trait affected by polygenic variation.

Despite these strengths, the RBr genetic repertoire lacks some important elements. All of the reported RBr mutations have been found to segregate independently of each other, so linkage analysis cannot be done with RBr. Also, none of the RBr loci used for education have been characterized at the molecular level. Therefore, we have sought to add such capabilities to RBr genetics, and our first step is to map the *anl *locus using molecular markers.

Although anthocyanin mutants are studied in *Brassica *and have been mapped in the related *Arabidopsis*, the *anl *locus has not been mapped in any *Brassica *species. In this study we create a microsatellite marker-based linkage map of *anl *in RBr. The linkage group may be integrated into known *B. rapa *linkage groups and used for comparative mapping among related species. Our findings open the door for experiments in linkage analysis and the use of molecular markers with RBr in science education.

## Results

### Microsatellite markers

We tested a total of 138 microsatellite markers for amplification and polymorphism in the RBr test population. Most (122 out of 138) were from *B. rapa*, but a few from other *Brassica *species were tested since the sequence homology between *Brassicas *in the U Triangle allowed for analysis of microsatellites first identified in *B. napus, B. oleracea *and *B. nigra *[16-18]. Of the 138 primer pairs tested, 37 amplified a product under our PCR conditions. Of these, 22 were polymorphic in our crosses (Table 1). We further tested these polymorphic microsatellites for linkage to *anl*.

**Table 1:** Microsatellite markers found to be polymorphic in RBr

Microsatellite	N^a^	Alleles^b^	Allele size (bp)^c^
*Bn9A*	31	2	200–205
*BRMS-006*	26	3	120–225
*BRMS-024 (b)*	72	3	170–200
*BRMS-033*	69	2	240–350
*BRMS-034*	47	3	150–180
*BRMS-037*	24	2	150–200
*BRMS-040*	89	2	195–270
*BRMS-042-2*	69	3	220–240
*BRMS-050*	73	2	170–190
*CAL-SSRLS-107*	52	3	140–165
*Na10-G10*	14	2	135–220
*Na12-H09*	44	3	130–215
*Ra2-A01*	55	3	95–120
*Ra2-D04*	38	2	160–170
*Ra2-E04*	24	3	110–120
*Ra2-E07*	48	4	90–185
*Ra2-G04*	43	3	180–195
*Ra2-G05*	56	3	130–175
*Ra2-G09*	14	2	235–245
*Ra2-D02B*	14	2	275–290
*Ra3-H09*	24	2	110–120
*Ra3-H10*	50	2	140–185

^a^Number of informative F_2 _plants used to test linkage.

^b^Number of alleles observed among the 177 *anl *plants constituting the RBr F_2 _test population.

^c^Approximate allele sizes based on visual comparison to 100-bp ladder on non-denaturing polyacrylamide minigels.

### F_2 _test population

We employed an inbred sib-pair design for genetic mapping, and chose plants for genotyping that would maximize the marker information obtained. Because rapid-cycling *Brassica rapa *strains are outbred, when a cross is conducted between two different strains, a given marker may be polymorphic between some pairs of parental generation plants but not others. Even for markers that are polymorphic between strains, some alleles may be shared. Therefore, after we had identified microsatellite markers that were polymorphic in the Standard *Brassica rapa *strain (Table 1), we tested them for polymorphism in each of the parental generation mating pairs. From these, we chose six mating pairs that displayed polymorphism for at least one marker. F_1 _generation plants were grown and siblings were mated (inbred sib-pairs). The F_1 _sib-pairs were surveyed, and those exhibiting a high degree of marker heterozygosity were chosen for further analysis. These 44 F_1 _plants (22 F_1 _sib-pairs) produced 699 F_2 _plants, of which 177 (25.32%) were anthocyaninless. This ratio is consistent with monogenic inheritance of this recessive trait. The 177 anthocyaninless F_2 _plants constituted the RBr test population for determining linkage between *anl *and the 22 polymorphic microsatellites (Table 1). The use of the inbred sib-pair design allowed us to clearly distinguish whether microsatellite bands detected in the F_2 _generation were identical by descent or identical by state.

### Linkage analysis and map construction

**Figure 1:**
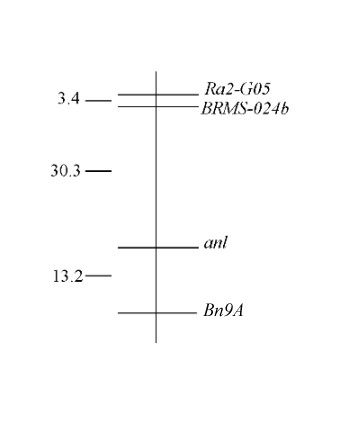
**Linkage map of the *anl *locus in RBr**. Map distances (cM) and locus order were evaluated using the Kosambi map function of Mapmanager QTX (p < 0.05, LOD ≥ 5.0) [19, 27].

After we identified markers that were polymorphic in the parental generation, we evaluated each as a candidate for linkage to the *anl *locus, followed by more detailed linkage analysis of candidates. All primer pairs used for mapping produced bands that segregated from each other as alleles, thus verifying that the microsatellites are single locus markers. We identified the linkage phase between *anl *and each marker in the F_1 _generation of each family and then classified anthocyaninless F_2 _progeny as either parental or recombinant. Three microsatellite markers were candidates for linkage to *anl *due to significant deviation from a 1:1 ratio of parental to recombinant genotypes. Through two-point LOD score analysis between *anl *and the candidate markers, we found that each presented a LOD score ≥ 4.6 at distances less than 34 cM from *anl*. Finally, we used multi-point LOD score analysis with Mapmanager QTX [19] to assemble a linkage group containing markers *Bn9A, BRMS-024b *and *Ra2-G05 *and the *anl *locus, all with LOD scores ≥ 5.0 (Table 2). In this linkage group (Figure 1), at least one marker is present on each side of *anl*. The discovery of microsatellite markers flanking *anl *is important in integrating this *anl *linkage group into known RBr linkage groups, and for comparative mapping studies.

**Table 2:** LOD scores and map distances for microsatellite loci linked to *anl *in RBr

Locus	Distance from *anl *(cM)^a^	LOD Score^a^
*Bn9A*	13.2	8.3
*BRMS-024b*	30.3	6.8
*Ra2-G05*	33.7	5.0

^a^Distances are based on the Kosambi map function using multi-point LOD analysis in Mapmanager QTX (p < 0.05) [19, 27]

We also tested all markers for pairwise linkage between markers, but did not find any linkage between those markers not linked to *anl*.

## Discussion

The genetic linkage of *anl *and *Bn9A *allows us to determine the chromosomal location of the *anthocyaninless *gene. *Bn9A *has been mapped to a region near the center of *B. rapa *linkage group R9 [20] (previously referred to as LG3 [21,22]). Thus, it can be inferred that both *anl *and the previously unmapped *Ra2-G05 *are also located on R9.

Our data strongly indicate that a polymorphic microsatellite that is amplified with primers for *BRMS-024 *[23] is part of the *anl *linkage group (Table 2) which we have shown to be a part of *Brassica rapa *linkage group R9 by virtue of its member *Bn9A*. However, *BRMS-024 *has been found by others to belong to linkage group R1 (G. Teakle, personal communication). Given such results, it is likely that sequences that can be amplified with *BRMS-024 *primers are present more than once in the *B. rapa *genome due to extensive intragenomic duplications [24]. Therefore, we refer to the locus that we have mapped as *BRMS-024b*.

Comparative mapping studies allow utilization of *B. rapa *map data in other crucifers. *B. rapa *and *Arabidopsis thaliana *are related by a common ancestor from which they differentiated ~14.5 to 20.4 million years ago [25]. Although markers may be twice as far apart in the larger *B. rapa *genome [22], the two species possess many regions with conserved organization; segments as large as 282.5 cM from the *B. rapa *map are observed in *Arabidopsis *[26]. A multinational effort to sequence the genomes of *Brassica *species is currently under way (Multinational *Brassica *Genome Project, 2007, http://www.brassica.info), including the sequencing of *B. rapa *linkage group R9 (Korean Brassica Genome Project, 2006, http://www.brassica-rapa.org). Linkage group R9, tentatively referred to as chromosome 5 [26], is now identified as cytogenetic Chr1 for use in comparative studies with the *Arabidopsis *chromosomal map [20]. The *Bn9A *locus on *B. rapa *Chr1 is located within a region that is conserved on *Arabidopsis *Chr1 (Korean *Brassica *Genome Project, 2006, http://www.brassica-rapa.org). Additionally, a small region just below the *Bn9A *locus in *B. rapa *is collinear with another region on *Arabidopsis *Chr1 (Korean *Brassica *Genome Project, 2006, http://www.brassica-rapa.org). While we cannot yet be certain that *anl *lies precisely within these conserved boundaries on *B. rapa *Chr1, the presence of the orthologous anthocyanin pigment gene *AN11 *on *Arabidopsis *Chr1 (TAIR accession number 2010356) supports the idea that the *Arabidopsis *ortholog of *anl *is located within the conserved regions.

## Conclusion

We have found the chromosomal location of the *anl *locus in RBr and identified three molecular markers linked to it. This linkage map of *anl *in *B. rapa *represents the first localization of an anthocyanin pigment gene in the *Brassicas*, and may be used for *B. rapa *map enhancement and comparative mapping of related species.

## Methods

### Polymerase chain reaction (PCR)

138 *Brassica *microsatellites and their PCR primer sequences were identified from published sources and primer pairs were produced by custom synthesis (Integrated DNA Technologies, Coralville, IA) (Table 3). Nucleotide sequences of primers were obtained from the *Brassica *microsatellite information exchange of the Multinational *Brassica *Genome Project web site http://www.brassica.info/ssr/SSRinfo.htm. All primer pairs were tested for ability to amplify a product from rapid-cycling *Brassica rapa *DNA under a standard set of PCR conditions. PCR reactions were carried out as follows: 1X Accuprime II buffer (Invitrogen Corporation, Carlsbad, CA), 1.5 mM MgCl_2_, 200 uM dNTPs, 0.05 U/uL Accuprime Taq DNA polymerase (Invitrogen), 40 ng *Brassica rapa *DNA, 10 pmol forward primer and 10 pmol reverse primer in a total reaction volume of 10 uL. The PCR program was as follows: 94°C for 2 minutes; 24 cycles at 94°C for 30 seconds; 61°C for 1 minute; 72°C for 1 minute; finished at 72°C for 4 minutes.

**Table 3:** Primer nucleotide sequences of polymorphic microsatellite markers in RBr

Microsatellite	Reference	Primer sequences^a^
*Bn9A*	[28]	GAGCCATCCCTAGCAAACAAG CGTGGAAGCAAGTGAGATGAT
*BRMS-006*	[23]	TGGTGGCTTGAGATTAGTTC ACTCGAAGCCTAATGAAAAG
*BRMS-024*	[23]	TGAATTGAAAGGCATAAGCA CAGCCTCCACCACTTATTCT
*BRMS-033*	[23]	GCGGAAACGAACACTCCTCCCATGT CCTCCTTGTGCTTTCCCTGGAGACG
*BRMS-034*	[23]	GATCAAATAACGAACGGAGAGA GAGCCAAGAAAGGACCTAAGAT
*BRMS-037*	[23]	CTGCTCGCATTTTTTATCATAC TACGCTTGGGAGAGAAAACTAT
*BRMS-040*	[23]	TCGGATTTGCATGTTCCTGACT CCGATACACAACCAGCCAACTC
*BRMS-042-2*	[23]	AGCTCCCGACAGCAACAAAAGA TTCGCTTCCTTTTCTGGGAATG
*BRMS-050*	[23]	AACTTTGCTTCCACTGATTTTT TTGCTTAACGCTAAATCCATAT
*CAL-SSRLS-107*	[29]	GTTAAGTGTGGCGTTAGAGG CCTTGGTACATGCCACTGAA
*Na10-G10*	[30]	TGGAAACATTGGTGTTAAGGC CATAGATTCCATCTCAAATCCG
*Na12-H09*	[30]	AGGCGTCTATCTCGAAATGC CGTTTTTCAGAATCTCGTTGC
*Ra2-A01*	[30]	TTCAAAGGATAAGGGCATCG TCTTCTTCTTTTGTTGTCTTCCG
*Ra2-D04*	[30]	TGGATTCTCTTTACACACGCC CAAACCAAAATGTGTGAAGCC
*Ra2-E04*	[30]	ACACACAACAAACAGCTCGC AACATCAAACCTCTCGACGG
*Ra2-E07*	[30]	ATTGCTGAGATTGGCTCAGG CCTACACTTGCGATCTTCACC
*Ra2-G04*	[30]	AAAACGACGTCATATTGGGC CGCTTCTTCTTCTCAGTCTCG
*Ra2-G05*	[30]	GCCAACTTAATTGATGGGGTC CCTCAATGTTCTCTCTCTCTCTCTC
*Ra2-G09*	[30]	ACAGCAAGGATGTGTTGACG GATGAGCCTCTGGTTCAAGC
*Ra2-D02B*	[30]	CACAGGAAACCGTGGCTAGA AACCCAACCTCAACGTCTTG
*Ra3-H09*	[30]	GTGGTAACGACGGTCCATTC ACCACGACGAAGACTCATCC
*Ra3-H10*	[30]	TAATCGCGATCTGGATTCAC ATCAGAACAGCGACGAGGTC

Nucleotide sequences of primers were from [31].

### DNA purification and quantitation

DNA was purified from frozen leaf tissue using Plant DNAzol Reagent (Invitrogen) with the manufacturer's recommended protocol, including the use of polyvinylpyrrolidone to remove polyphenolics. The concentration of DNA samples was assayed using Quant-iT PicoGreen Reagent (Invitrogen).

### Gel electrophoresis for genotyping

Microsatellite alleles were resolved by non-denaturing polyacrylamide gel electrophoresis. Most PCR products were resolved using minigels (Mini-Protean III (Bio-Rad Laboratories, Hercules, CA)) consisting of 8% acrylamide/bis (24:1) run at 150 V for 60 to 90 minutes. When marker alleles could not be resolved on minigels, they were resolved in large (18 cm) gels (Protean II (Bio-Rad Laboratories)) which consisted of 8% acrylamide/bis (19:1) run at 150 V for 1350 Volt-hours. Gels were stained with SYBR Green Stain (Invitrogen) and visualized with a Molecular Dynamics Storm 860 Scanner (GE Healthcare, Piscataway, NJ).

### Genetic crosses and data analysis

Three-generation pedigrees were used to assess linkage between the *anl *locus and microsatellite markers. The rapid-cycling *Brassica rapa *for the parental generation were strains of Wisconsin Fast Plants obtained from Carolina Biological Supply Company (Burlington, North Carolina). The parental generation consisted of a true breeding anthocyaninless strain ("Non-Purple Stem, Hairless," catalog number 15-8812) and a true-breeding purple strain ("Standard *Brassica rapa*," catalog number 15-8804). One anthocyaninless and one purple plant constituted a mating pair. F_1 _sibling pairs were then crossed to produce the F_2 _generation.

Before genotyping families, microsatellite markers were screened for usefulness by testing each pair of primers for the ability to amplify a product and identify polymorphism in DNA of a panel of several Standard *Brassica rapa *plants. Each mating pair in the parental generation was then tested for polymorphism for those markers. When a given marker was found to be polymorphic in a pair of parental generation plants, their F_1 _progeny were genotyped for that marker. Finally, for each pair of mated F_1 _siblings in which a given marker was polymorphic and informative, their F_2 _generation offspring with anthocyaninless phenotype were genotyped for that marker. This inbred sib-pair mating design ensured that alleles from the F_2 _anthocyaninless test population could be traced to their parental generation ancestor, so that linkage phase of the marker and *anl *loci would be known.

For each marker that was polymorphic within a family, anthocyaninless F_2 _offspring were assigned a parental or recombinant designation for segregation between *anl *and the marker. The parental:recombinant ratio was then assessed for deviation from the expected 1:1 ratio for unlinked loci by the chi-square test. Markers presenting significant chi-square values (p < 0.05) were identified as candidates for linkage to *anl*, and two-point LOD analysis was performed between the marker and *anl*. Those markers with preliminary LOD scores greater than 3.0 were further analyzed with Mapmanager QTX (Kosambi map function, p < 0.05) to determine marker arrangements and multi-point LOD scores [19,27].

## Competing interests

The author(s) declares that there are no competing interests.

## Authors' contributions

CB participated in the design of the study, carried out all breeding and genotyping, conducted analysis of the data, and drafted the manuscript. DW participated in the conception of the project and design of the study, assisted with analysis of the data, and edited the manuscript.

Both authors read and approved the final version of the manuscript.
